# Mechanical Properties and Liquid Absorption of Calcium Phosphate Composite Cements

**DOI:** 10.3390/ma16165653

**Published:** 2023-08-17

**Authors:** Ioana Lacan, Marioara Moldovan, Codruta Sarosi, Stanca Cuc, Mihaela Pastrav, Ioan Petean, Razvan Ene

**Affiliations:** 1 Department of Physics and Chemistry, Technical University of Cluj-Napoca, 400114 Cluj-Napoca, Romania; ioana_lacan@yahoo.com; 2 Department of Polymer Composites, Raluca Ripan Institute for Research in Chemistry, Babeș-Bolyai University, 30 Fantanele Street, 400294 Cluj-Napoca, Romania; marioara.moldovan@ubbcluj.ro (M.M.); codruta.sarosi@ubbcluj.ro (C.S.); 3 Department of Orthodontics, “Iuliu Hatieganu” University of Medicine and Pharmacy, 31 Avram Iancu Street, 400117 Cluj-Napoca, Romania; 4 Faculty of Chemistry and Chemical Engineering, University Babes-Bolyai, 11 Arany János Street, 400028 Cluj-Napoca, Romania; ioan.petean@ubbcluj.ro; 5 14 Department, Orthopedics, Anesthesia and Intensive Care, University of Medicine and Pharmacy “Carol Davila”, 020021 Bucharest, Romania; razvan77ene@yahoo.com; 6 Orthopedics and Traumatology Department, Bucharest Emergency University Hospital, 050098 Bucharest, Romania

**Keywords:** calcium phosphate, chitosan, composite cements, mechanical properties

## Abstract

Calcium phosphate cements present increased biocompatibility due to their chemical composition being similar to that of the hydroxyapatite in the hard tissues of the living body. It has certain limitations due to its poor mechanical properties, such as low tensile strength and increased brittleness. Thus, the optimal way to improve properties is through the design of novel composite cements. The purpose was fulfilled using a 25% hydroxyethyl methacrylate (HEMA) mixed with 3% urethane dimethacrzlate (UDMA) base matrix with various ratios of polyethylene glycol (PEG 400) and polycaprolactone (PCL). Mineral filler is based on tricalcium phosphate (TCP) with different chitosan ratio used as bio-response enhancer additive. Four mixtures were prepared: S0—unfilled polymer matrix; S1 with 50% TCP filler; S2 with 50% chitosan + TCP filler; and S3 with 17.5% chitosan + TCP mixed with 17.5% nano hydroxyapatite (HA). The mechanical properties testing revealed that the best compressive strength was obtained by S2, followed by S3, and the worst value was obtained for the unfilled matrix. The same tendency was observed for tensile and flexural strength. These results show that the novel filler system increases the mechanical resistance of the TCP composite cements. Liquid exposure investigation reveals a relative constant solubility of the used filler systems during 21 days of exposure: the most soluble fillers being S3 and S2 revealing that the additivated TCP is more soluble than without additives ones. Thus, the filler embedding mode into the polymer matrix plays a key role in the liquid absorption. It was observed that additive filler enhances the hydrophobicity of UDMA monomer, with the matrix resulting in the lowest liquid absorption values, while the non-additivated samples are more absorbent due to the prevalence of hydrolytic aliphatic groups within PEG 400. The higher liquid absorption was obtained on the first day of immersion, and it progressively decreased with exposure time due to the relative swelling of the surface microstructural features. The obtained results are confirmed by the microstructural changes monitored by SEM microscopy. S3 and S2 present a very uniform and compact filler distribution, while S1 presents local clustering of the TCP powder at the contact with the polymer matrix. The liquid exposure revealed significant pore formation in S0 and S1 samples, while S3 and S2 proved to be more resistant against superficial erosion, proving the best resistance against liquid penetration.

## 1. Introduction

Bone cements are polymer-based materials used for bone reconstruction and for hip and joint anchorage during orthopedic interventions. These materials have been in continuous development since 1980, aiming for mechanical property improvement and biocompatibility increase [1,2]. Bone cements are grouped based on their chemical composition into: acrylic bone cements (ABC) [3] and calcium phosphate cements (CPC) [4]. Also, they can be classified according to their orthopedic and dental applications.

ABCs provide fast attachment of the implant to the bone’s hard tissue and are predominantly used in orthopedic surgery for joint anchorage and replacement. They present several disadvantages, such as an increased temperature caused by the exothermic reaction during cement curing [5], a lack of bioactivity [6], and monomer cytotoxicity [7].

CPCs are bioresorbable and biocompatible and are used predominantly in maxillofacial surgery due to their low mechanical strength [8]. The mechanical properties of CPC bone cements could be increased by the development of calcium sulfate systems with better binding properties, good biocompatibility, and a resorption rate of 2 months [9]. These materials have some limitations regarding their osteoconductivity and faster resorption, creating an acid environment in the tissue surrounding the implant, which further creates mechanical instability [10].

Calcium phosphates have been used for bone cement composition improvement in recent decades. The chemical composition of CPC is similar to that of natural bone, meaning that Ca^2+^ and PO^3−4^ ions released may promote osteoconductivity and osteogenesis [11]. Calcium phosphates provide a controllable degradation rate depending on the Ca/P ratio and filler type [12]. Since their first usage in dentistry (1970) and orthopedics (1980), they have gained special interest as bone graft replacements in periodontal and osteochondral regeneration and as potential carriers in controlled release systems [4,13,14]. The inorganic salts have been added to improve the biological characteristics of bone cements, which also increase their mechanical properties and reduce the polymerization process’s heat [15,16].

Chitosan (CS) is a potential additive for bone cement improvement because of its specific characteristics, such as biocompatibility, biodegradability, supports bone regeneration, and less cytotoxicity [17]. Therefore, it is widely used in orthopedic applications, providing support for bone regeneration [18]. It also presents intra-molecular hydrogen bonds that assure good heat resistance, making it an optimal ingredient to be used in bone cements compared to other specific materials such as collagen, gelatin, and alginate.

CS is the second most abundant natural polysaccharide on earth (found in the shells of snails, crabs, shrimps, and insects) [19]. It is a copolymer of glucosamine and N-acetyl glucosamine linked by β 1–4 glucoside bonds. Its sensitivity at high pH values has led to the use of this biopolymer in nanoparticle drug delivery systems. Moreover, CS-based materials are ideal bioactive materials due to their special properties such as antibacterial, anti-fungal, hydrophilicity, biodegradability, and their ability to stimulate cell adhesion, which facilitates their proliferation and thus increases their viability [20,21]. Unfortunately, the high CS amount introduced in bone cements considerably reduces their mechanical properties, restricting their application with increased mechanical loads.

Thus, the mechanical and biological performance might be adjusted by a proper dosage of the filler components and polymer matrix. Urethane dimethacrylate (UDMA) and 2-hydroxyethyl methacrylate (HEMA) polymers have a large variety of medical and dental applications. UDMA monomers generate a relatively rigid hydrophobic polymer, providing high mechanical properties to the cement, while HEMA forms a highly flexible hydrogel, which can act as a reservoir for drug delivery. Likewise, polycaprolactone (PCL) is a biodegradable, biocompatible, and bioactive material, considering the possibility of replacing bone tissue due to its unique properties given by its easy availability, cost-effectiveness, and modification suitability [22].

Bone cements behavior in a liquid environment is very important for clinical applications; for example, water absorption may affect their microstructure and thus cause a strong decrease in their mechanical properties due to the bonding agents hydrolyzing, which further generates internal microcracks. Thus, the liquid absorption behavior is an important investigation that should be performed regarding the new bone cement formulation. It should be evaluated immediately after the polymerization process and verified at regular intervals up to 21 days [23]. Water penetration respects the diffusion laws, and hydrophilic monomers facilitate the internal spreading. Data in the literature shows that the hydrophilic behavior might be controlled by a proper adjustment of the powder/liquid ratio [23].

Deb et al. [23,24] studied the water absorption characteristics of hydroxyapatite (HA)-reinforced bone cements, reporting that HA introduction reduces the water absorption of the bone cement. The effect is enhanced by a silane coupling agent applied to the HA filler. Recent studies provide evidence of bone cement applications such as medical treatment in osteoporotic vertebral compression fractures in the elderly population. These cements require an increased and efficient expansion at contact with body fluid, which is achieved by a porous structure obtained by the introduction of a hydrophobic monomer in the composition [25].

The aim of the present article is the investigation of the novel bone cement formulation using a complex filler system based on TCP additive with chitosan regarding the improvement of mechanical properties and liquid absorption. UDMA hydrophobic monomer is intended for the control of the polymer matrix hydrophilicity and to act as a liquid penetration moderator. 

## 2. Materials and Methods

### 2.1. Materials

The polymeric materials used in current research are UDMA, HEMA, PEG 400, PCL, and Chitosan; all were purchased from Sigma-Aldrich, Darmstadt, Germany as analytical purity. Mineral fillers TCP and HA were also purchased from Sigma-Aldrich, Darmstadt, Germany. A chemical polymerization system was used for cement hardening, implying that the paste obtained by mixing the two phases is divided into two equal proportions: one in which the polymerization initiator POB (benzoyl peroxide) is added in the proportion of 2% of the paste and the other in which the polymerization accelerator DHEPT (dihydroxy-ethyl-p-toluidine) is dissolved in the proportion of 0.75% of the paste. The polymerization occurs when both pastes are mixed in equal proportion, leading to the formation of the hardened material. Thus, the final product presents a single polymer phase representing the composite matrix, which physico-chemically bonds the filler particles. The composition of the prepared samples is described in Table 1. 

### 2.2. Mechanical Properties Testing

Materials mechanical properties influence their ability to be worked into a suitable shape and the final product’s resistance under working solicitation. Therefore, it is very important to know what the mechanical solicitations of the bone cement are after in vivo insertion. As previously known, tensile strength shows the material behavior at axial elongation; compressive strength shows the behavior under axial compressive solicitation; and flexural strength reveals the bending behavior of the material under tangential solicitations [26]. The Young modulus gives a characterization of the elastic properties of the investigated samples and is usually calculated from the tensile strength testing curve [26]. Thus, mechanical property testing was conducted as follows.

#### 2.2.1. Compressive Strength (According to ISO 4049/2000) [27]

Two molds were used for the sample preparation: the first with a diameter of 40 mm and a height of 80 mm, and the second mold with a diameter of 30 mm and a height of 60 mm. The composite was molded and polymerized until its complete solidification, and samples were further extracted and investigated. Tensile strength measurement was effectuated on an Lloyds Instron Universal Analyzer (Lloyd Instrumente, Ameteklns, West Sussex, England, UK) testing machine. Force and elongation were digitally measured by the testing machine computer-aided system. All tests were performed at 23 °C, and the final values are the mean of 10 punctual determinations.

#### 2.2.2. Tensile Strength (According to ADA, Sp.27) [28]

Tensile strength was measured using the diametric compression test (an indirect test for determining tensile strength). The disc sample is compressed across its diameter in the appropriate device until it is fractured, with the dimensions of the disc being 0.3 cm thick and 0.6 cm in diameter. The specimens are subjected to compression along the cylinder generator. The force F = 5 N acting on the cylinder clamped between the plates of the apparatus, which acts at a speed of 1 mm/min, causes tensile stresses to occur in the plane of the vertical diameter. The tensile strength was given by the average of 10 determinations.

#### 2.2.3. Flexural Strength (According to ISO 4049/2000) [27]

The flexural strength has been achieved by the 3-point technique, reflecting the stiffness and resistance capacity of materials to deformation or breakage due to mechanical stress. The specimens used in this test had a parallelepiped shape, i.e., 2.5 cm long, 0.2 cm thick, and 0.2 cm deep. To determine the flexural strength, the specimens are symmetrically supported on two 2 mm diameter supports, with the distance between the axes of the two supports being l = 20 mm. The bending force F (N) acts centrally on the specimen by means of a 2 mm diameter cylinder at a speed of 0.5 mm/minute and a force of 0.5 N.

### 2.3. Liquid Absorption and Solubility Testing

Absorption and solubility evaluations are carried out according to ISO 4049/2000 [27]. Ten cement discs (n = 10) of each investigated material were made, using a Teflon matrix with a diameter of 151 mm and a thickness of 1 mm. The matrix was filled with the sample and covered with glass by pressing so as to obtain a smooth and homogeneous surface of hardened material. After the polymerization time had elapsed (2–3 min after mixing the two pastes), the discs were stored individually in a drying cabinet at 23 °C until a constant mass (M1) was obtained by weighing. After this step, the samples were individually immersed in flasks of distilled water at 37 °C for 21 days. After a certain time interval (1 day, 3 days, 7 days, 14 days, and 21 days), the discs were removed from the immersion medium and gently dried with absorbent paper. Each disc was then weighed using an analytical balance with an accuracy of 0.001 g (Ohaus Explorer), obtaining the mass M2. The weighed discs were stored for 2 h in a shaker until a constant M3 table was obtained. The absorption and solubility of these materials was calculated using the formulas [24]:

(1) $$W_{p}(\%)=\frac{M_{2}-M_{1}}{M_{1}}\times 100$$

and

(2) $$W_{l}(\%)=\frac{M_{1}-M_{2}}{M_{1}}\times 100$$

where *W_p_* represent the absorption of the samples and *W_l_* represent the solubility of the samples.

### 2.4. Statistical Analysis

Data were subjected to ANOVA and Tukey’s test for post hoc comparisons between groups, and the significance level was set at *p* = 0.05 using Origin2019b Graphing & Analysis software (2019). For each investigated material property, 10 samples (n = 10) were taken.

### 2.5. X-ray Diffraction (XRD)

X-ray diffraction was effectuated with an X-ray diffractometer, the XRD-6000 Shimadzu (produced by Shimadzu Company, Tokyo, Japan), and a Bragg Brentano goniometer using monochromatic Cuk_α_ radiation with wavelength λ = 1.540560. The investigation was conducted from 10 to 70 degrees 2 theta. The peaks were identified using Match 1.0 software equipped with the PDF2 database package, Crystal Impact Company, Bonn, Germany.

### 2.6. Scanning Electron Microscopy (SEM) Investigation

Scanning electron microscopy was used to investigate the microstructure of the composites tested for absorption and solubility. The investigation of the samples was carried out with FEI’s INSPECT S electron microscope, Hillsboro, OR, USA. The samples were analyzed immediately after the polymerization reaction and after 21 days of immersion in distilled water at a temperature of 37 °C. They were inspected without metallization at an acceleration voltage of 30 kV, and secondary electron (SEI) images were recorded with high resolution.

## 3. Results and Discussion

### 3.1. Mechanical Properties

Both dental and orthopedic hard tissues are subjected to complex mechanical solicitation due to the specific movements of the body parts, such as chewing for dental or walking for orthopedic situations. Thus, the real complex mechanical solicitation could be decomposed using the parallelogram rule for the mechanics into the axial solicitation (where the force acts longitudinally on the sample), i.e., compression and tensile (elongation), and into the tangential solicitation (where the force acts perpendicularly on the sample), causing bending solicitation that is better described by flexural strength testing.

**Figure 1:**
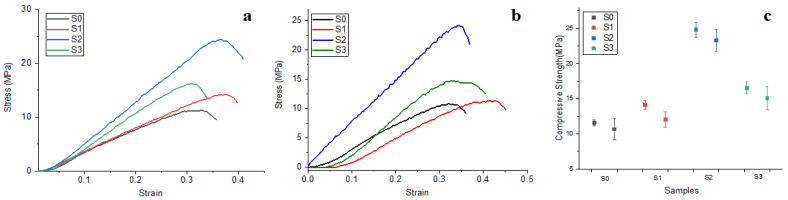
Compressive strength curves obtained for the samples with diameters of (**a**) 40 mm, (**b**) 30 mm, and (**c**) a value variation plot.

The compressive strength testing curves are presented in Figure 1. There were bigger samples (e.g., 40 mm diameter) in Figure 1a and smaller ones (e.g., 30 mm diameter) in Figure 1b. Both situations reveal that compressive strength increases in the following order: S0 < S1 < S3 < S2. 

It results that S3 presents the best compression strength (Figure 1c) due to the optimal composition of the TCP filler additived with the proper chitosan amount; this fact is supported by the literature data [29]. Also, S3 presents excellent compressive strength due to the synergy involved between TCP—HA—CS. Statistical analysis indicates that no significant difference occurs between S3 and S2 samples (*p* > 0.05). On the other side, S1, having the same TCP content as S3 but without CS, has a low compressive strength (almost half of the value obtained by S3) and is situated closely to the unfilled sample S0, the statistical difference being insignificant (*p* > 0.05). Thus, there are two statistical groups: the first belongs to the samples with high compression strengths S3 and S2 and the other group is represented by the samples with low compression strengths S1 and S0. The ANOVA test followed by the Tukey post hoc test revealed a significant statistical difference between those two groups (*p* < 0.05). It clearly demonstrates the benefits brought on by the chitosan addition of tricalcium phosphate filler particles.

**Figure 2:**
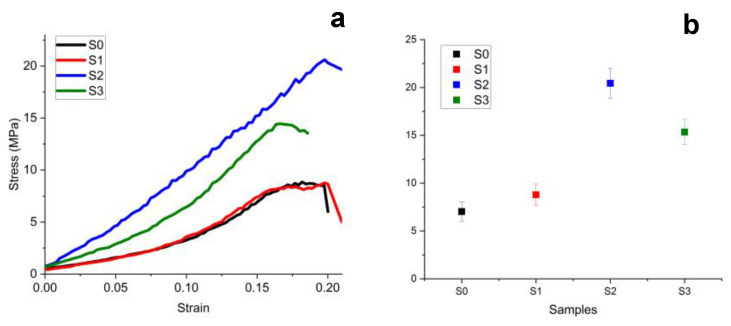
Tensile strength results: (**a**) experimental curve and (**b**) mean values with standard deviation.

Tensile strength testing curves are presented in Figure 2a. The general aspect of the curves resulted for S2 and S3 samples has a typical shape for a tenacious material with a prolonged linear zone of the curve showing good elastic properties and less elongation until failure. Samples S0 and S1 present curve allure typical for a ductile material with low loading force that causes significant strain followed by great elongation until the final failure.

The best stress results were recorded for S2 cement, 20.43 MPa, followed by S3 > S1 > S0, Figure 2b. As can be seen, the stress-strain curve of S0 cement overlaps S1, emphasizing once again that the hardening and storage time do not influence the properties of these materials. Thus, the statistical analysis reveals two groups: the first one is formed by S0 and S1 samples with low tensile strength (*p* > 0.05), and the second group is represented by the higher values of tensile strength formed by S2 and S3 (*p* > 0.05). ANOVA statistics followed by the Tukey post hoc test reveal significant statistical differences between those two groups (*p* < 0.05). The results prove that TCP additived with chitosan assures the best resistance on the tensile component of the complex solicitation of the implants. 

**Figure 3:**
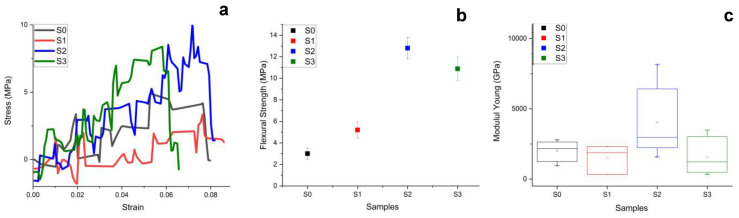
Flexural strength results: (**a**) experimental curves, (**b**) mean values, and (**c**) Young Modulus.

Flexural strength testing curves are presented in Figure 3a, and the mean values are displayed in Figure 3b.

The experimental curves in Figure 3a present a wide variation of the stress value along with strain-increasing facts that evidence a nonlinear distribution of the internal tension through the sample microstructure. The polymeric matrix tends to resist overtangential solicitations that are unevenly dissipated through those filler particles. Thus, the chitosan-additivated samples present better behavior, resisting higher stress values than un-additivated samples. The situation is more evident on the mean values plot in Figure 3b. The higher bending strength was obtained for S2 = 12.1 MPa, followed closely by S3 = 10.37 MPa, while the un-additivated samples present flexural strength around 4 MPa in good agreement with literature [30,31]. There are two statistical groups: low flexural strength (samples S0 and S1 *p* > 0.05) and high flexural strength (samples S2 and S3 *p* > 0.05. A significant statistical difference was observed between those two groups’ *p* < 0.05, as resulted after ANOVA and Tukey post hoc tests. It clearly shows that chitosan addition to the filler composition improves the samples behavior under tangential solicitations. This fact is also supported by the Young Modulus (Figure 3c), which shows that the S2 sample has the best elastic properties, followed closely by S3. Unfortunately, S1 Young Modulus is situated below unfilled polymer sample S0, proving that filling with unadditived TCP decreases the composite elastic properties. Another factor that can justify a lower Young’s modulus at S1 compared to S0 is the size of the filler, the type and shape of the fillers that couple effectively or not to the matrix, and also the presence of pores in the polymerized sample [32]. The microstructural unevenness affecting the stress dissipation within the microstructure also affects the elastic properties of the samples being subjected to a non-uniform elastic deformation based on the assumption of stress equilibrium in the sample requiring a certain dispersion time [25,33,34].

### 3.2. Liquid Absorption and Solubility

**Figure 4:**
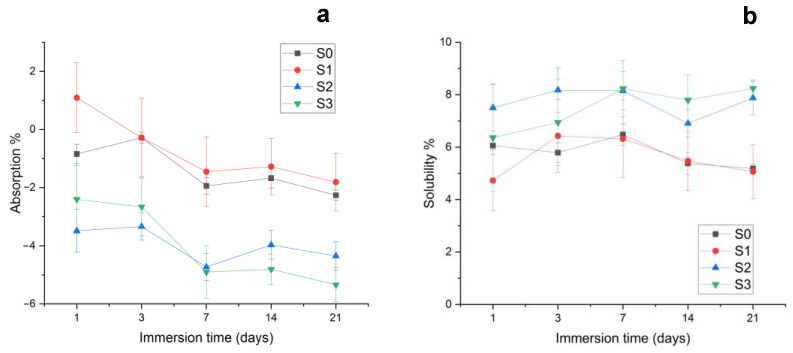
Sample exposure to the liquid environment: (**a**) Liquid absorption and (**b**) solubility.

Previous studies have shown that filler particles and binding agents added to the polymer matrix alter liquid absorption [35]. The Wp and Wl values in Equation (1) show that cements with a higher filler ratio have lower liquid absorption (Figure 4a). Therefore, liquid absorption is situated as follows: S2 < S3 < S0 < S1 after the first day of immersion. This hierarchy is maintained up to 21 days of liquid exposure, and a slightly decreased tendency is observed due to the relative swelling of the already infiltrated layers. 

The liquid absorption strongly depends on the PEG 400 diluting monomer, which contains a hydrolytic aliphatic group [36], which facilitates the liquid diffusion through the polymer matrix. The opposite UDMA monomer is known for its hydrophobic behavior [37], and it might moderate the liquid absorption within the polymer matrix. A slightly increased amount of UDMA in S3 might sustain a relative hydrophobicity increase in this sample. The other samples have the same UDMA content, and thus S2 features the best resistance to water penetration. It is a strong proof that there exists an active synergy between the UDMA hydrophobic monomer and the chitosan additivation of the filler.

On the other side, filler particle distribution within the matrix and its relative solubility might affect liquid absorption [38]. Figure 4b shows that mineral fractions within composites present increased solubility, while low-filled composites and unfilled matrix are less soluble. Well-dispersed nanostructured HA generates a dense and compact composite that is very resistant to liquid penetration [39]. Thus, mineral loss is to occur only on the composite surface, while the deeper layers remain intact due to the perfect insulation of filler particles by the excellent lamination of the polymer matrix.

### 3.3. Crystalline Matter Assesment

**Figure 5:**
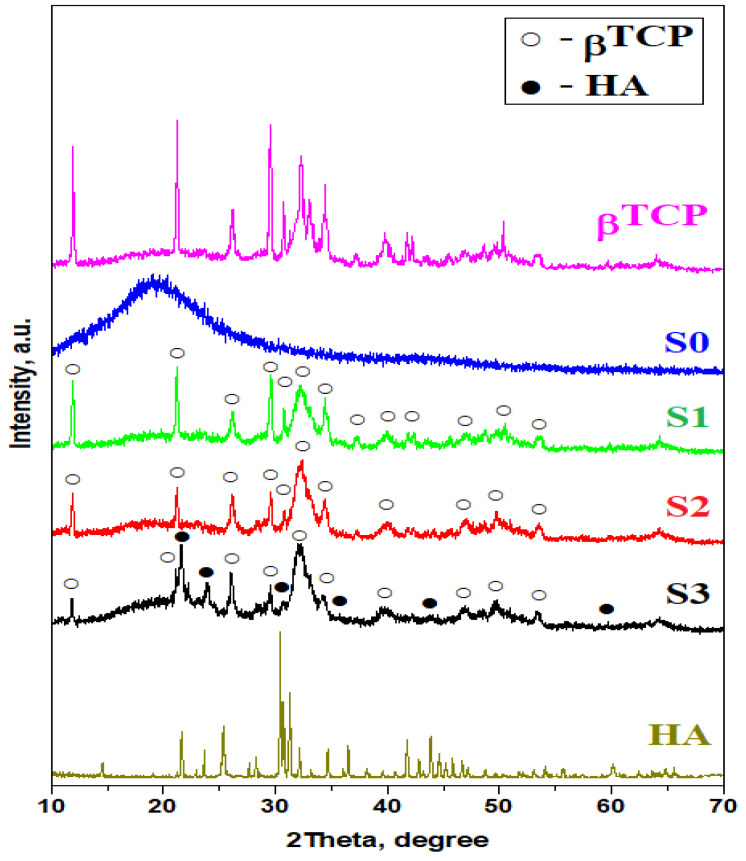
X-ray diffraction patterns for the composite samples and for the raw filler powders β-TCP and HA.

The filler raw powders were investigated by X-ray diffraction for their mineral composition assessment (Figure 5). Tricalcium phosphate powder proves to be β-TCP all peaks identified in the XRD pattern belong to JCPDS 70-2065. The peaks are well developed, proving the high crystallinity of the powder related to the rhombohedral elementary cell, but are also slightly broadened due to the nanocrystallite presence.

Hydroxyapatite powder used as filler was also subjected to the XRD investigation. The obtained pattern presented in Figure 5 reveals well-developed peaks that are slightly broadened, a fact related to the presence of nano-crystallites. All obtained peaks belong to the hydroxyapatite crystallized in the hexagonal system according to JCPDS 72-1243.

The crystalline matter within composite samples was investigated with XRD, and the obtained results are presented in Figure 5. The S0 sample presents a typical amorphous allure due to the lack of mineral filler. Samples S1–S3 present relevant diffraction peaks corresponding to the mineral filler. Multiple peaks of convolution are observed at 23.04 degrees 2 theta. Merged peaks belong to β TCP, and they are most likely due to the amorphization effect induced by the admixture of nanocrystalline filler with amorphous polymer.

The S1 sample presents well-developed diffraction peaks, indicating a significant presence of crystalline matter. They are rather broadened than narrow, and their relative intensities are somehow lower than for a typical crystalline material. It indicates a wide dispersion of nanoparticles into an amorphous polymer matrix. All obtained peaks belong to β calcium triphosphate (β TCP) corresponding to PDF: 01-086-1585. The crystallite size was determined using Scherrer formula, resulting in about 60 nm.

Figure 5 shows that the S2 sample is very similar to the S1 sample; all observed peaks correspond to β TCP. The peaks relative intensities are lower than observed for S1 due to the lower amount introduced in the composite mixture. The Scherrer formula applied to the most representative β TCP peak indicates a nanocrystallite size of 64 nm.

The S3 sample evidences both peaks for β TCP and for hydroxyapatite. The dominant peak belongs to the hydroxyapatite, indicating that it is the dominant mineral in the composition, followed closely by the β TCP. The most relevant peak of HA is much broadened, and thus applying the Scherrer formula results in an average crystallite diameter of about 40 nm. This fact is very useful for dental materials due to its dimensional compatibility with the HA nanostructures within human enamel.

### 3.4. Microstructural Aspects

**Figure 6:**
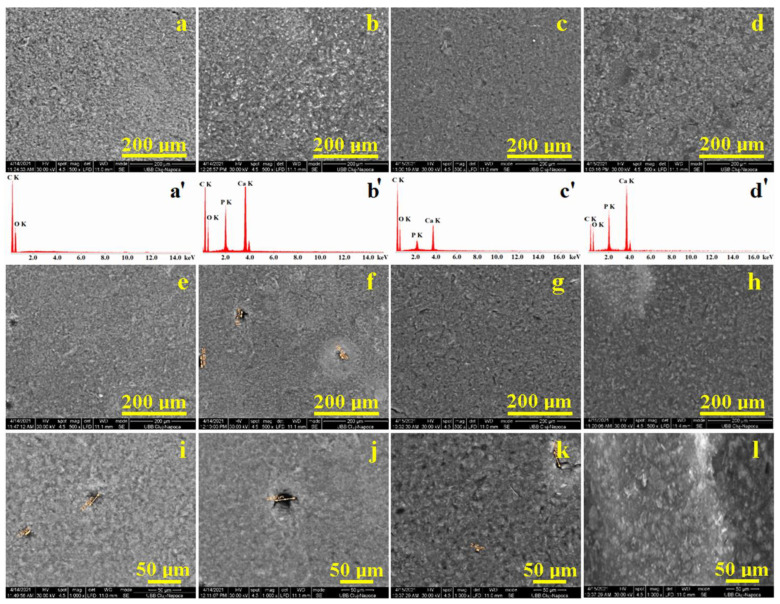
SEM images of the sample’s microstructure: initial: (**a**) S0, (**b**) S1, (**c**) S2, (**d**) S3, and after 21 days of immersion in liquid: (**e**) S0, (**f**) S1, (**g**) S2, (**h**) S3, and high magnification of the microstructure after 21 days of immersion: (**i**) S0, (**j**) S1, (**k**) S2, and (**l**) S3. EDX spectra are presented for images a’, b’, c’, and d’.

The microstructural aspects were observed by SEM microscopy (Figure 6). The initial unexposed samples were investigated first. Unfilled sample S0 presents a very uniform and compact microstructure of polymer-rounded clusters having a mean diameter of 5 ± 2.5 μm (Figure 6a). The corresponding EDX spectrum reveals only carbon and oxygen belonging to the polymeric mixture. The addition of TCP filler modified the sample microstructure as observed for S1, Figure 6b. Tricalcium phosphate fine particles are very well mixed with the polymer matrix, generating a compact physical mixture characterized by a uniform distribution of fine microstructural clusters with a rounded shape and diameter of about 10 ± 3.2 μm. The presence of TCP clusters was also evidenced by the EDX spectrum in Figure 6b. It indicates that the polymer matrix realizes optimal physical lamination of the filler particles. 

The addition of TCP + chitosan filler (S2 sample) induces significant microstructural modification (Figure 6c). Filler particles are more efficiently dispersed into the polymer matrix, and the clusters are the finest, with rounded shapes and a diameter of about 3 ± 1.5 μm. The presence of the mineral filler particles is also sustained by Ca and P amounts, as evidenced by the EDX spectrum in Figure 6c. They are very well embedded into the polymer, which indicates an extra chemical bond beyond the physical lamination. Thus, chitosan acts as a mediator between TCP fine particles and UDMA hydrophobic monomers within the matrix.

The S3 sample presents a heterogeneous microstructure due to the complex filler system based on both TCP and nano HA mediated by chitosan additive, a fact supported by the EDX spectrum in Figure 6d. Thus, two types of grains appear: polyhedral grains with very fine and compact microstructure inside them (these belong to the nano HA mixture with the polymer matrix, a fact sustained by the HA crystallites measured from the XRD pattern) and the clustered microstructure typical for TCP based on rounded clusters of about 3 ± 1.8 μm that are very well attached to the neighboring grains because of chitosan mediation between filler particles and UDMA hydrophobic monomers. The facts agree with the literature data [40,41].

EDX analysis brings important information regarding the filler distribution in the composite cement’s structure. The obtained values are summarized in Table 2. Thus, we observe that S0 contains only carbon and oxygen. The other samples contain P and Ca amounts that bear a close resemblance to the stoichiometry ratio within the chemical formula of the fillers and their introduced quantities.

The higher amount of Ca and P was identified in sample S1, while the lower amount was identified in sample S2. This fact corresponds to the composite recipe presented in Table 1 and finally proves that the filler was properly incorporated into the polymer matrix.

The liquid exposure for 21 days had a significant influence on the S0 and S1 samples, which evidenced superficial pores formation. Two rounded pores of about 10 μm diameter are observed in the left side of the observation field of the image in Figure 6e, indicating that the PEG 400 hydrophilic monomers in S0 sample generate local weakening of the polymer matrix. The situation is more prone for the S1 sample, where due to the hydrophilic monomers within the matrix, the pore occurrence is associated with local mineral loss, and thus the diameter increases at about 25 ìm. Such mineral losses are reported in the literature at the composite surface contact with liquid due to the local penetration between polymer and filler particles, which starts delamination that finally results in particle loss [42,43]. Chitosan additived samples S2 and S3 keep their microstructure almost intact, preventing water infiltration and observable mineral loss; no pores are visible in Figure 6g,h.

High magnification SEM images of S0 and S1, Figure 6i,j, reveal better the pores occurrence caused by the liquid infiltration propagated on the hydrophilic monomer within PEG 400. There is also a weakening of the polymer matrix clusters due to the prolonged exposure to the liquid environment. On the other side, the high magnification observation of S2 sample, Figure 6k, reveals the excellent cohesion of the polymer matrix with the TCP clusters. There are only a few small pores of about 5 μm in diameter formed by the dissolution of exposed filler particles in the outermost layer of the composite. In addition to this minor superficial erosion, there are no signs of liquid infiltration, proving the synergy between chitosan and the UDMA hydrophobic monomer. The situation is very similar for the S3 sample, as observed in Figure 6l. TCP grains become more evident that the fine HA-based grains due to their relative interaction with prolonged exposure to the liquid environment. 

## 4. Conclusions

Chitosan additivation to TCP filler particles proves to be very effective at the composite microstructural level, mediating their adhesion to the polymer matrix. A synergic effect appears between chitosan and the UDMA hydrophobic monomer within the matrix, assuring better cohesion than the simple physical lamination that occurs in the unadditivated samples. Thus, the best mechanical properties were obtained for chitosan-additivated samples while unadditived ones presented poor values, especially at flexural strength. The efficacy of chitosan-mediated bonding of the filler particles assures optimal protection against liquid penetration into the deeper layers and prevents in-depth filler particle dissolution.

## Figures and Tables

**Table 1:** Bone cement samples composition.

Cement	Organic Phase (%)				Inorganic Phase (%)			Initiation System (%)	
	UDMA	HEMA	PEG 400	PCL	TCP	Chitosan	HA	POB	DHEPT
S0	3	25	22	0	0	0	0	2	0.75
S1	3	25	22	0	50	0	0	2	0.75
S2	3	25	22	0	37.5	12.5	0	2	0.75
S3	5	25	20	15	0	17.5	17.5	2	0.75

UDMA: 1,6-bis(methacryloxy-2-ethoxycarbonylamino)-2,4,4-trimethylhexane; HEMA: 2-hydroxyethyl methacrylate; PEG 400: Polyethylene glycol with a low-molecular-weight (380–420 g/mol); PCL: Polycaprolactone; TCP: Tricalcium phosphate; HA: Hydroxyapatyte; POB: Benzoyl peroxide; DHEPT: dihydroxy-ethil-p-toluidine.

**Table 2:** Elemental composition of the cement samples.

Samples	Identified Elements, Wt.%.			
	C	O	P	Ca
S0	63.54	36.46	-	-
S1	35.6	22.5	16.76	25.14
S2	44.12	26.37	10.75	18.76
S3	39.18	24.56	13.79	22.47

## Data Availability

Data are available upon request from the corresponding authors.

## References

[1] Lukina Y., Safronova T., Smolentsev D., Toshev O. (2023). Calcium Phosphate Cements as Carriers of Functional Substances for the Treatment of Bone Tissue. Materials.

[2] Koons G.L., Diba M., Mikos A.G. (2020). Materials design for bone-tissue engineering. Nat. Rev. Mater..

[3] Valencia Zapata M.E., Mina Hernandez J.H., Grande Tovar C.D. (2020). Acrylic Bone Cement Incorporated with Low Chitosan Loadings. Polymers.

[4] Xu H.H.K., Wang P., Wang L., Bao C., Chen Q., Weir M.D., Chow L.C., Zhao L., Zhou X., Reynolds M.A. (2017). Calcium phosphate cements for bone engineering and their biological properties. Bone Res..

[5] Robu A., Antoniac A., Grosu E., Vasile E., Raiciu A.D., Iordache F., Antoniac V.I., Rau J.V., Yankova V.G., Ditu L.M. (2021). Additives Imparting Antimicrobial Properties to Acrylic Bone Cements. Materials.

[6] Eil Bakhtiari S.S., Karbasi S., Hassanzadeh Tabrizi S.A., Ebrahimi-Kahrizsangi R., Salehi H. (2020). Evaluation of the effects of chitosan/multiwalled carbon nanotubes composite on physical, mechanical and biological properties of polymethyl methacrylate-based bone cements. Mater. Technol..

[7] De Mori A., Di Gregorio E., Kao A.P., Tozzi G., Barbu E., Shangani-Kerai A., Draheim R.R., Roldo M. (2019). Antibacterial PMMA Composite Cements with Tunable Thermal and Mechanical Properties. ACS Omega.

[8] Alkhasawnah Q., Elmas S., Sohrabi K., Attia S., Heinemann S., El Khassawna T., Heiss C. (2021). Confirmation of Calcium Phosphate Cement Biodegradation after Jawbone Augmentation around Dental Implants Using Three-Dimensional Visualization and Segmentation Software. Materials.

[9] Liu J., Wang Y., Liang Y., Zhu S., Jiang H., Wu S., Ge X., Li Z. (2023). Effect of Platelet-Rich Plasma Addition on the Chemical Properties and Biological Activity of Calcium Sulfate Hemihydrate Bone Cement. Biomimetics.

[10] Fernandez de Grado G., Keller L., Idoux-Gillet Y., Wagner Q., Musset A.M., Benkirane-Jessel N., Bornert F., Offner D. (2018). Bone substitutes: A review of their characteristics, clinical use, and perspectives for large bone defects management. J. Tissue Eng..

[11] Furko M., Balázsi K., Balázsi C. (2023). Calcium Phosphate Loaded Biopolymer Composites—A Comprehensive Review on the Most Recent Progress and Promising Trends. Coatings.

[12] Chen X., Li H., Ma Y., Jiang Y. (2023). Calcium Phosphate-Based Nanomaterials: Preparation, Multifunction, and Application for Bone Tissue Engineering. Molecules.

[13] Pujari-Palmer M., Guo H., Wenner D., Autefage H., Spicer C.D., Stevens M.M., Omar O., Thomsen P., Edén M., Insley G. (2018). A Novel Class of Injectable Bioceramics That Glue Tissues and Biomaterials. Materials.

[14] Chow L.C. (2009). Next generation calcium phosphate-based biomaterials. Dent. Mater. J..

[15] Fang C.H., Lin Y.W., Sun J.S., Lin F.H. (2019). The chitosan/tri-calcium phosphate bio-composite bone cement promotes better osteo-integration: An in vitro and in vivo study. J. Orthop. Surg. Res..

[16] Vach Agocsova S., Culenova M., Birova I., Omanikova L., Moncmanova B., Danisovic L., Ziaran S., Bakos D., Alexy P. (2023). Resorbable Biomaterials Used for 3D Scaffolds in Tissue Engineering: A Review. Materials.

[17] López-Valverde N., Aragoneses J., López-Valverde A., Rodríguez C., Macedo de Sousa B., Aragoneses J.M. (2022). Role of chitosan in titanium coatings. trends and new generations of coatings. Front. Bioeng. Biotechnol..

[18] Devernois E., Coradin T. (2023). Synthesis, Characterization and Biological Properties of Type I Collagen–Chitosan Mixed Hydrogels: A Review. Gels.

[19] Islam M.M., Shahruzzaman M., Biswas S., Nurus Sakib M., Rashid T.U. (2020). Chitosan based bioactive materials in tissue engineering applications-A review. Bioact. Mater..

[20] Bakshi P.S., Selvakumar D., Kadirvelu K., Kumar N.S. (2020). Chitosan as an environment friendly biomaterial–a review on recent modifications and applications. Int. J. Biol. Macromol..

[21] Supernak M., Makurat-Kasprolewicz B., Kaczmarek-Szczepañska B., Pałubicka A., Sakowicz-Burkiewicz M., Ronowska A., Wekwejt M. (2023). Chitosan-Based Membranes as Gentamicin Carriers for Biomedical Applications—Influence of Chitosan Molecular Weight. Membranes.

[22] Dwivedi R., Kumar S., Pandey R., Mahajan A., Nandana D., Katti D.S., Mehrotra D. (2020). Polycaprolactone as biomaterial for bone scaffolds: Review of literature. J. Oral Biol. Craniofac. Res..

[23] Pascual B., Gurruchaga M., Ginebra M.P., Gil F.J., Planell J.A., Gon I. (1999). Influence of the modification of P/L ratio on a new formulation of acrylic bone cement. Biomaterials.

[24] Deb S., Braden M., Bonfield W. (1995). Water absorption characteristics of modified hydroxyapatite bone cements. Biomaterials.

[25] Chen L., Tang Y., Zhao K., Yu X., Yao B., Li X., Zha X., Zhang B., Tan Q., Yang Z. (2022). Self-expanding PMMA composite bone cement with sustained release of gentamicin sulfate and alendronate using water absorption pathways. Mater. Des..

[26] Murugan S.S. (2020). Mechanical Properties of Materials: Definition, Testing and Application. Int. J. Mod. Stud. Mech. Eng..

[27] (2000). Dentistry—Polymer-Based Filling, Restorative and Luting Materials.

[28] (2016). Polymer-Based Restorative Materials.

[29] Redondo F.L. (2022). Preparation of Porous Poly (Lactic Acid)/Tricalcium Phosphate Composite Scaffolds for Tissue Engineering. Biointerface Res. Appl. Chem..

[30] Seuba J., Maire E., Adrien J., Meille S., Deville S. (2021). Mechanical properties of unidirectional, porous polymer/ceramic composites for biomedical applications. Open Ceram..

[31] Mosallanezhad P., Nazockdast H., Ahmadi Z., Rostami A. (2022). Fabrication and characterization of polycaprolactone/chitosan nanofibers containing antibacterial agents of curcumin and ZnO nanoparticles for use as wound dressing. Front. Bioeng. Biotechnol..

[32] Pal R. (2005). Porosity-dependence of effective mechanical properties of pore-solid composite materials. J. Compos. Mater..

[33] Cahyanto A., Liemidia M., Karlina E., Zakaria M.N., Shariff K.A., Sukotjo C., El-Ghannam A. (2023). Bioactive Carbonate Apatite Cement with Enhanced Compressive Strength via Incorporation of Silica Calcium Phosphate Composites and Calcium Hydroxide. Materials.

[34] Wronski S., Wit A., Tarasiuk J., Lipinski P. (2022). The impact of the parameters of the constitutive model on the distribution of strain in the femoral head. Biomech. Model. Mechanobiol..

[35] Islam S., Nassar M., Elsayed M.A., Jameel D.B., Ahmad T.T., Rahman M.M. (2023). In Vitro Optical and Physical Stability of Resin Composite Materials with Different Filler Characteristics. Polymer.

[36] Sarosi C., Moldovan M., Soanca A., Roman A., Gherman T., Trifoi A., Chisnoiu A.M., Cuc S., Filip M., Gheorghe G.F. (2021). Effects of Monomer Composition of Urethane Methacrylate Based Resins on the C=C Degree of Conversion, Residual Monomer Content and Mechanical Properties. Polymers.

[37] Fonseca A.S.Q.S., Moreira A.D.L., de Albuquerque P.P.A.C., de Menezes L.R., Pfeifer C.S., Schneider L.F.J. (2017). Effect of monomer type on the CC degree of conversion, water sorption and solubility, and color stability of model dental composites. Dent. Mater..

[38] Boboia S., Moldovan M., Prejmerean C., Sarosi C., Roman A., Ardelean I. (2015). Influence of initiation system and filler ratio on the properties of new flowable dental composites. Mater. Plast..

[39] González Ocampo J.I., Escobar Sierra D.M., Ossa Orozco C.P. (2016). Porous bodies of hydroxyapatite produced by a combination of the gel-casting and polymer sponge methods. J. Adv. Res..

[40] Ardelean A.I., Dragomir M.F., Moldovan M., Sarosi C., Paltinean G.A., Pall E., Tudoran L.B., Petean I., Oana L. (2023). In Vitro Study of Composite Cements on Mesenchymal Stem Cells of Palatal Origin. Int. J. Mol. Sci..

[41] Lukina Y., Bionyshev-Abramov L., Kotov S., Serejnikova N., Smolentsev D., Sivkov S. (2023). Carbonate-Hydroxyapatite Cement: The Effect of Composition on Solubility In Vitro and Resorption In Vivo. Ceramics.

[42] Bandeira Lopes L., Paredes F., Pimenta A., Carpinteiro I. (2020). Management of an Unsuccessful Regenerative Endodontic Procedure after Tooth Fracture: A Case Report. Dent. J..

[43] Sequeira D.B., Seabra C.M., Palma P.J., Cardoso A.L., Peça J., Santos J.M. (2018). Effects of a New Bioceramic Material on Human Apical Papilla Cells. J. Funct. Biomater..

